# Surface topography changes and wear resistance of different non-metallic telescopic crown attachment materials in implant retained overdenture (prospective comparative in vitro study)

**DOI:** 10.1186/s12903-024-04839-w

**Published:** 2024-09-26

**Authors:** Sherif M. Abdel Hamid, Rim A. Selima, Mohamed Z. Basiony

**Affiliations:** Prosthodontics Department, Faculty of Dentistry, Pharos University, Alexandria, Egypt

**Keywords:** Implant-retained overdenture, Telescopic attachment, PEEK, Zirconia, Cobalt-chrome, Wear resistance

## Abstract

**Background:**

The purpose of this study was to evaluate the effect of using different types of metallic and non-metallic telescopic crown attachment materials on wear resistance and surface tomography changes in implant-retained mandibular overdentures.

**Materials and methods:**

Completely edentulous mandibular epoxy models were fabricated, in which two implants were placed in the canine region and retained to the implants with three different material combinations used for the construction of telescopic attachments. Thirty-three identical mandibular overdentures were fabricated using the conventional standardized technique. The study groups were divided into three categories according to the material used for the construction of the secondary copings. The primary copings in all the study groups were constructed of PEEK, while the secondary coping in group I was PEEK, group II was ZrO_2_ and CoCr for group III. Primary copings were cemented on a ready-made abutment. Secondary copings were placed over the primary copings in the desired path of insertion, then picked up into the intaglio surface of the overdentures. A cyclic loading machine was used to apply repeated insertion-removal cycles simulating nearly 10 years of clinical use. Stereomicroscope with a built-in camera was used to monitor the reduction in width of the primary copings to evaluate the wear resistance of each material combination.

**Results:**

There was highly statistically significant difference between the study groups after the application of 1.000, 5.000 and 10.000 cycles. The highest level of wear resistance was recorded for the PEEK/PEEK combination, whereas PEEK/ZrO_2_ and PEEK/CoCr showed no significant differences.

**Conclusions:**

Implant retained overdenture with PEEK-PEEK telescopic crown attachment is associated with the highest wear resistance among all the study groups. PEEK-PEEK combination may be the treatment of choice for fabrication of telescopic attachment in implant retained overdenture as it provides better resistance to wear. It offers the advantages for geriatric patients as it decreases the possibility for repeated repair and replacement of attachment, increase long-term patient satisfaction and shelf life of prosthesis.

## Introduction

Since 1989, telescopic attachments have been utilized to anchor overdentures in the rehabilitation of edentulous mandibles. In the case of a severely atrophied edentulous mandible, two implants placed in the canine region with strong telescoping attachments for overdenture retention proved to be a viable and efficient therapeutic option with long-term success [1].

Historically, telescopic crown attachments were fabricated from cobalt-chromium. However, research has linked between the presence of corrosion products around the implant leading to peri-implantitis. The alternative materials for double crowns include other metals such as base metal alloys and titanium owing to their differing properties and potential to reduce corrosion-related complications. Nevertheless, reported sensitivities to certain of these metals necessitate the use of non-metallic substitutes. Some individuals demonstrated nickel sensitivity and, to a lesser extent, cobalt sensitivity [2].

The retention of double-crown-retained prostheses is influenced by factors such as crown tapers, crown heights, the materials used, and friction between the axial walls of the inner and outer crowns [3]. However, owing to the wear between materials, retention may diminish with time [4]. Advancements in the development of dental materials have been tremendous, including ceramics and high-performance polymers such as PEEK and zirconia, which offer new possibilities for durable and biocompatible telescopic crowns. However, each innovative dental material has been compared with other well-established materials, including metal alloys, over the past many years [5].

Typically, a material with a high resistance to wear is selected as the primary crown, whereas a more flexible material is used for the secondary crown [6]. Clinicians and manufacturers are investigating alternatives for double-crown-retained overdentures that are less costly, more aesthetically appealing, biocompatible, and offer comparable precision and long-term retention.

The ceramic materials used for the fabrication of telescopic attachments were first described in 2000 [7]. Zirconia is non-corrosive, and its color mimics that of teeth, with excellent biocompatibility, superior mechanical strength, and wear resistance compared with precious alloys. In this regard, the materials utilized for double-crown systems have a substantial effect on their retention [8].

Poly-ether-ether-ketone (PEEK) was produced by modifying poly-ether-aryl-ketone, the primary thermoplastic high-performance polymer group. Both PEEK and ZrO_2_ have superior biocompatibility and may be used for a variety of dental applications, including provisional abutments, dental implants, and fixed dental prostheses (FDPs) [9]. According to reports, PEEK is noted for its biocompatibility and suitability for various dental applications, including double crown systems [10]. The innovative combination of these two biocompatible materials, PEEK and ZrO_2_, aimed to create non-metallic telescopic crowns.

PEEK was implemented as the primary coping because it produces no corrosion products, outstanding chemical resistance, as well as resistance to thermal and post-irradiation degradation, are conferred by its structure. Compared to advanced aesthetic computer-aided design/computer-aided manufacturing (CAD/CAM) polymers, PEEK is chemically inert and exhibits a reduced solubility and water absorption. Owing to its encouraging physico-mechanical characteristics, PEEK exhibits some benefits over conventional alloys and ceramic dental materials [11–13].

Compared to other retainers, PEEK cause less trauma and distribute occlusal stresses along the abutment long axis. PEEK has cushion properties that decreases the forces over the implant and hence improve crestal bone loss. Also, PEEK has less plaque accumulation that decreases periapical gingival inflammation. Due to the mentioned reasons, PEEK was chosen to be used as primary coping [14].

Therefore, this in vitro study aims to assess the surface topography and wear resistance of three different non-metallic combinations used in constructing telescopic attachments for future overdentures. The null hypothesis posits that the material combinations do not influence the wear resistance of the telescopic attachments.

## Materials and methods

Different types of attachment materials have been used to provide better retention and stability, although some materials exhibit significant changes in the surface topography and continuous wear after the application of an occlusal load, with subsequent loss of fit, which affects the denture retention over time. Therefore, this prospective comparative study was conducted to evaluate the effectiveness of different material combinations on the resistance to wear when constructing telescopic attachments to support an implant retained overdenture.

The sample size was calculated at the 0.05 significance level via R software, with a pooled standard deviation of 6.85 and a 95% Confidence interval. The total sample size was thirty-three mandibular overdenture samples. The models were coded and randomly divided into three groups, each group received 11 models (*N* = 11) [15].

The study groups were divided according to the materials used for the fabrication of secondary crowns, while all the primary copings were fabricated from PEEK [16]. Group I involved PEEK secondary coping, Group II involved ZrO_2_ secondary coping and Group III involved CoCr secondary coping.

### Fabrication of mandibular replica

By using a transparent epoxy resin (Bond Quick 5, Konishi Co., Osaka, Japan) in accordance with the manufacturer’s instructions, the resin was rapidly poured into a prefabricated rubber silicon (polyester Resin, Nells Materials Co., UK) mould with the help of vibrator machine to prevent air bubbles from emerging into the final model. The epoxy resin model was removed from the mould to serve as a mandibular edentulous replica.

### Denture construction

Visible-light-cure-acrylic resin (VLC) (RMH3 Dental, Chantilly, Virginia) was used to fabricate a trial denture base onto which an occlusion rim was attached due to its ease manipulation and handling. Anatomic (30°) teeth were set in place giving due care to occlusal plane height such that the teeth did not exceed half the retromolar pad region. Wax contouring (festooning) was carried out on the trial denture giving due care to the shape of the polished surfaces. Denture was flasked, invested in a three-pour of plaster and stone. Wax elimination through boil-out and then heat cure Poly-Methyl-Methacrylate (PMMA) (Acrostone Co Ltd) was mixed according to manufacturer’s instructions, packed, pressed, processed and deflasked accordingly. Finally, the denture was finished and polished. Thirty-three identical complete mandibular overdentures were fabricated following the same conventional standardized techniques.

### Surgical guide fabrication

**Fig. 1:**
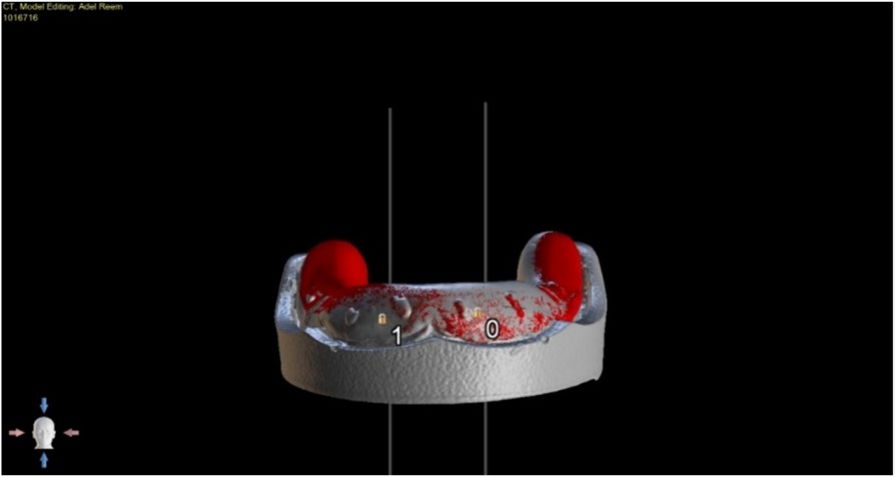
Surgical guide planning

**Fig. 2:**
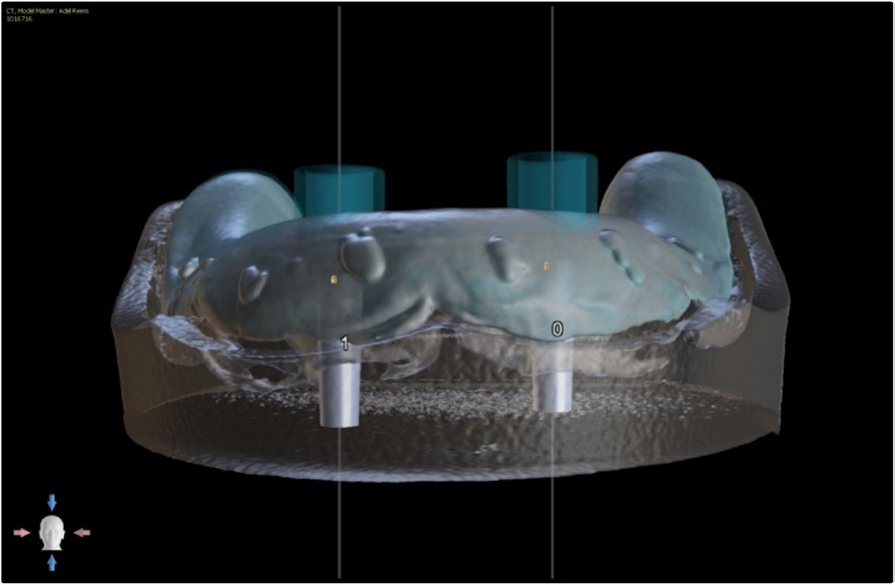
Planning of implant position using OnDemand3D Software

**Fig. 3:**
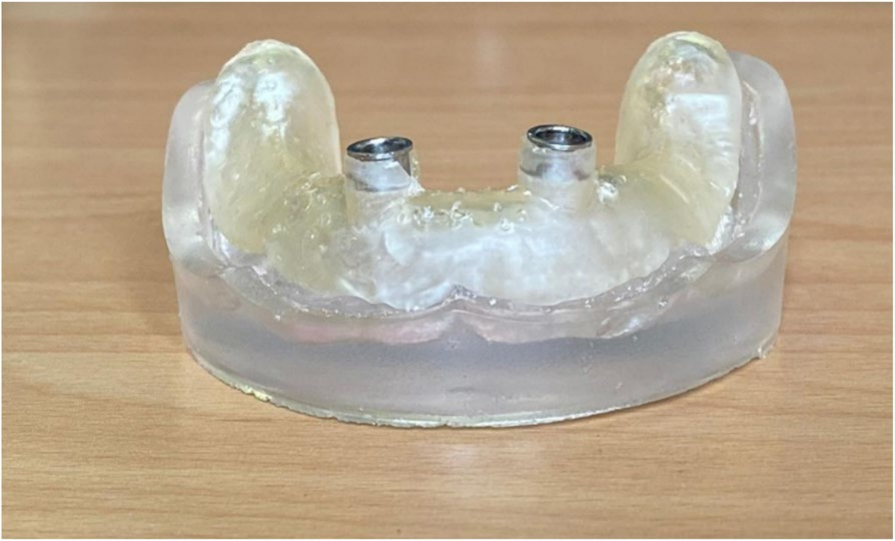
Surgical guide with two metal holes placed over the planned implants' sites

The overdenture was modified to be used as a radiographic surgical template by attaching gutta-percha cones (Meta biomed, Korea) to the mandibular canine region on both sides. A virtual radiographic scan of the model was obtained via CBCT (J. Morita, Veraview R100, Japan) to obtain the Dicom series, which was analyze using OnDemand3D software (OnDemand3D, Cybermed Inc., Seoul, Korea) The series was segmented, and a 3D model was generated in STL format (Figs. 1 and 2). A stereolithographic partially guided surgical guide was fabricated from transparent acrylic resin (Nissin Dental Products Inc., Kyoto, Japan) using CAD/CAM system and printed via a Formlab 3D printer (Formlabs, Somerville, MA, USA), in which two metal holes were placed over the planned implant sites (Fig. 3).

### Implant placement in the epoxy-resin models

Two implants were placed bilaterally at the planned implant sites using serial drills (2 to 3.3 mm drills, respectively) to the correct depth indicated on the drills up to reach a depth of 10 mm. The implants were placed at zero tilt indicating that the path of insertion and removal parallel to the implants’ long axis resulting in decreased lateral destructive forces on the implant. A mandibular two-implant overdenture is considered the standard treatment for edentulous patients [17, 18].

A countersink drill was used to flare the implant site allowing easy insertion of the implants into its prepared sites and simulating their clinical use intra-orally. Then two implants (Vitronex V-line Implant, Vitronex Co. Ltd., Italy) with a diameter of 3.3 mm and length of 10 mm were installed, and a primary stability of 35 Ncm was achieved.

**Fig. 4:**
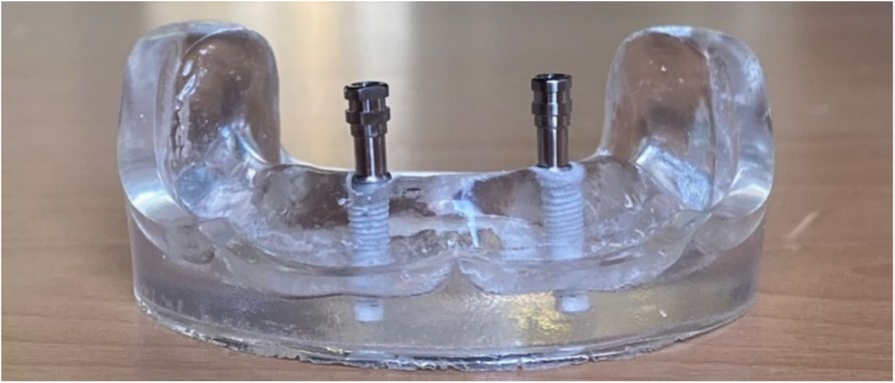
Implant abutments placement

The implant abutments were subsequently screwed to the implants using a torque wrench up to reach 20 Ncm torque to be ready for the future modifications (Fig. 4).

### Implant abutments preparation and attachment fabrication

**Fig. 5:**
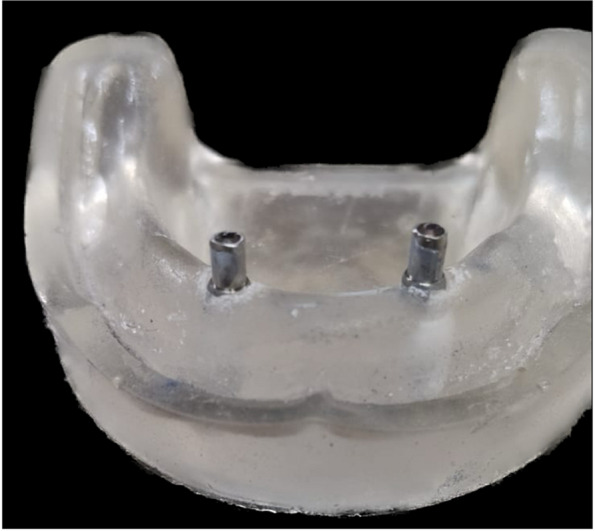
Implant abutments’ preparation

Ready-made abutments (Vitronex V-line Implant, Vitronex Co. Ltd., Italy) with a 1.2 mm shoulder and a 6° axial taper was reduced to a 4 mm axial height (Fig. 5). To minimize reflection, each model was sprayed with a thin coating of anti-reflection scan spray (Shera Scan Spray) before scanning. The prepared abutments were next scanned with a laboratory scanner (InaEos X5 extraoral scanner, SIRONA, Germany).

**Fig. 6:**
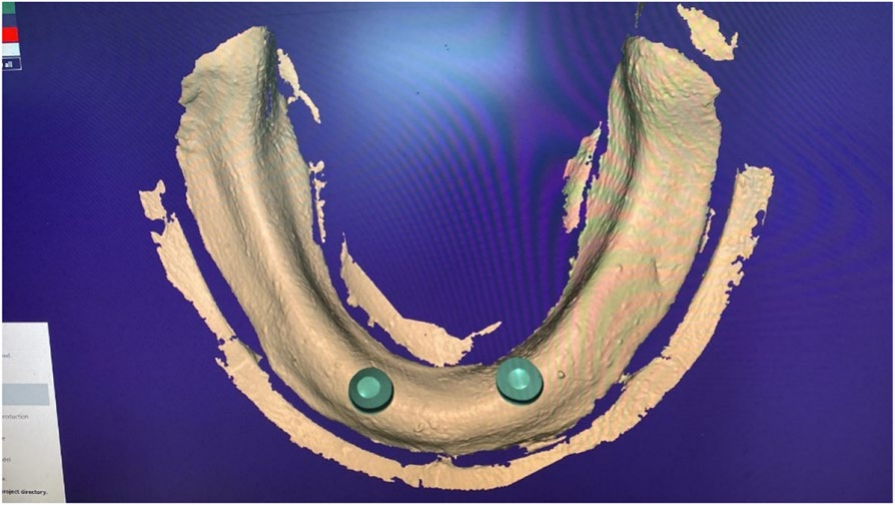
Designing of the primary crowns

Primary copings were designed to form a cylindrical pattern keeping a common path of placement considered, with a 6° axial taper to ensure resiliency of the attachment, and thickness of 2 mm occlusally, 1.5 mm axially and 1.2 mm shoulder [19]. The primary copings were then milled (Formlabs, Somerville, MA, USA) from Poly-Ether-Ether-Ketone (PEEK) (CeraDirect, Hong Kong) (Fig. 6).

### Surface treatment of the PEEK primary coping and the implant abutments

**Fig. 7:**
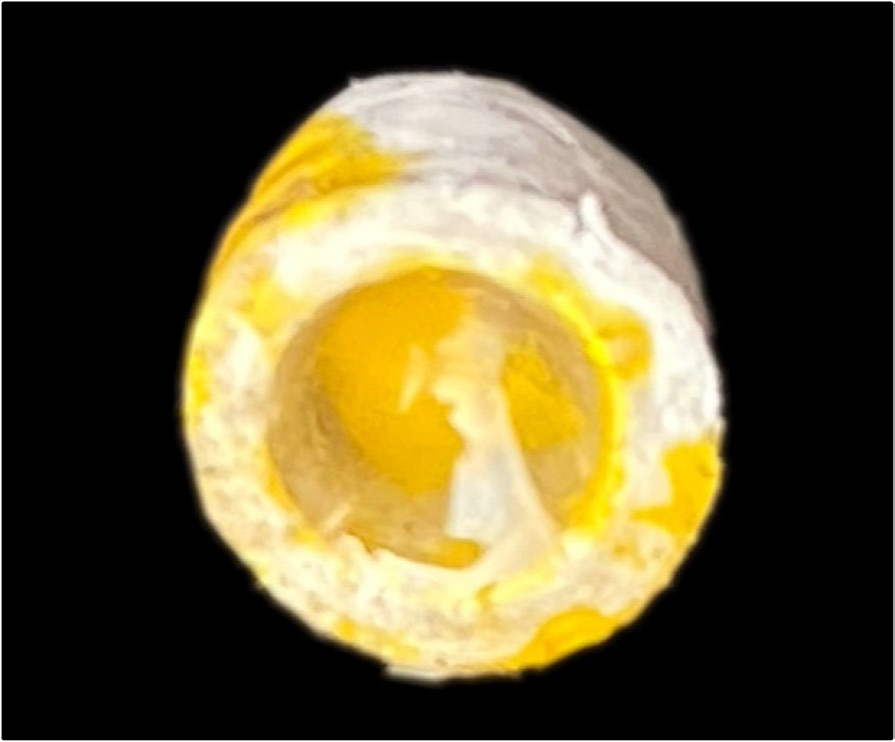
Surface treatment of the inner surface of PEEK primary coping with 98% sulfuric acid

The inner surface of the PEEK primary copings as well as the outer surface of the implant abutments were sandblasted with 50 μm grain size particles for 15 s of aluminum oxide at 0.2 MPa (3 bar pressure, distance of 1 cm, 15 times), creating a microrough surface and enhance the bonding strength [20]. The inner surface of PEEK was treated with 98% sulfuric acid for 30 s to create a microroughened surface and to allow the adhesion with the cement material (Fig. 7) [21, 22].

### Cementation of the PEEK primary coping over the implant abutments

The holes of the implant abutments were sealed using temporary restoration (MD Temp hydraulic temporary restoration, Meta Biomed, kirea) to protect the abutment screw.

**Fig. 8:**
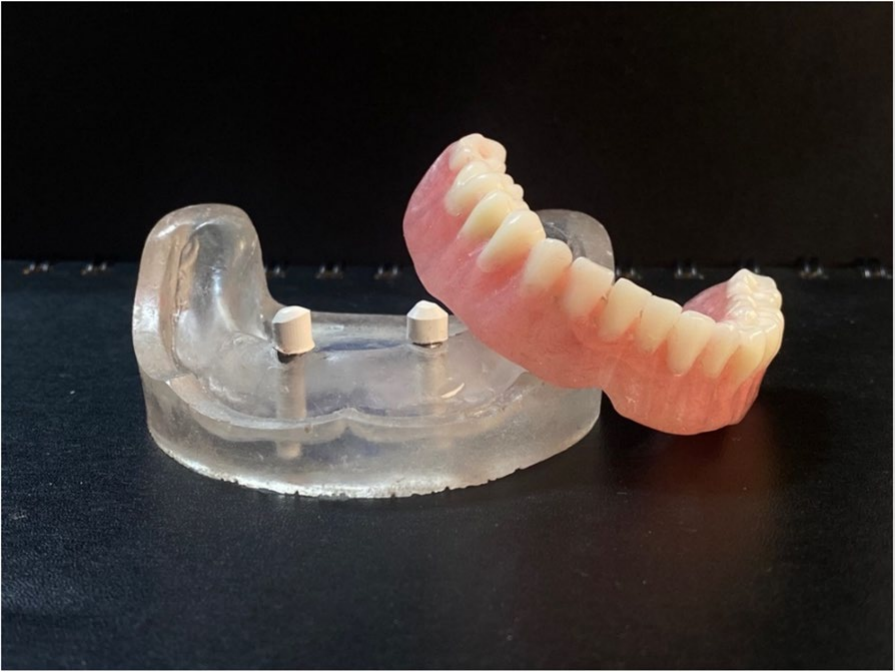
PEEK primary coping cementation over the abutment

Dual-cure resin cement was used according to the manufacturer’s instructions, and Hancem resin cement (Handae Han, Handae, Korea) was used for cementation of the PEEK primary coping over the implant abutments (Fig. 8).

### Fabrication of secondary copings

**Fig. 9:**
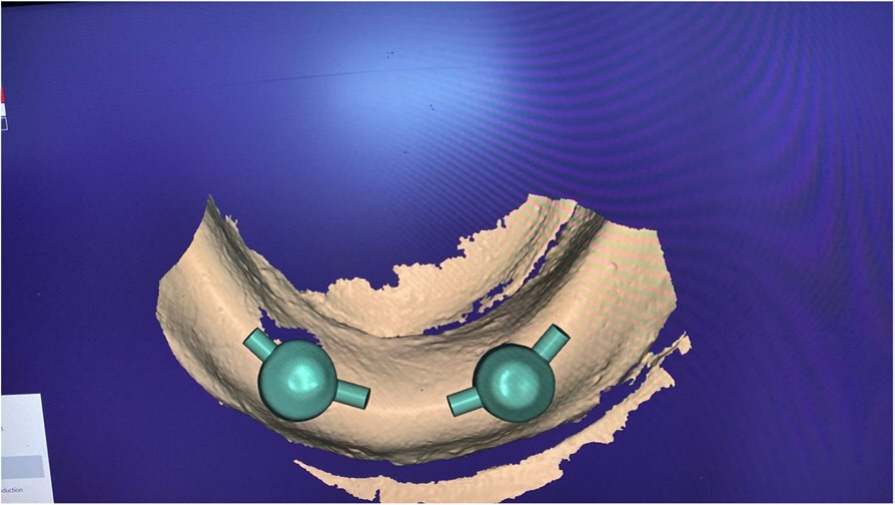
Designing of the secondary crowns

**Fig. 10:**
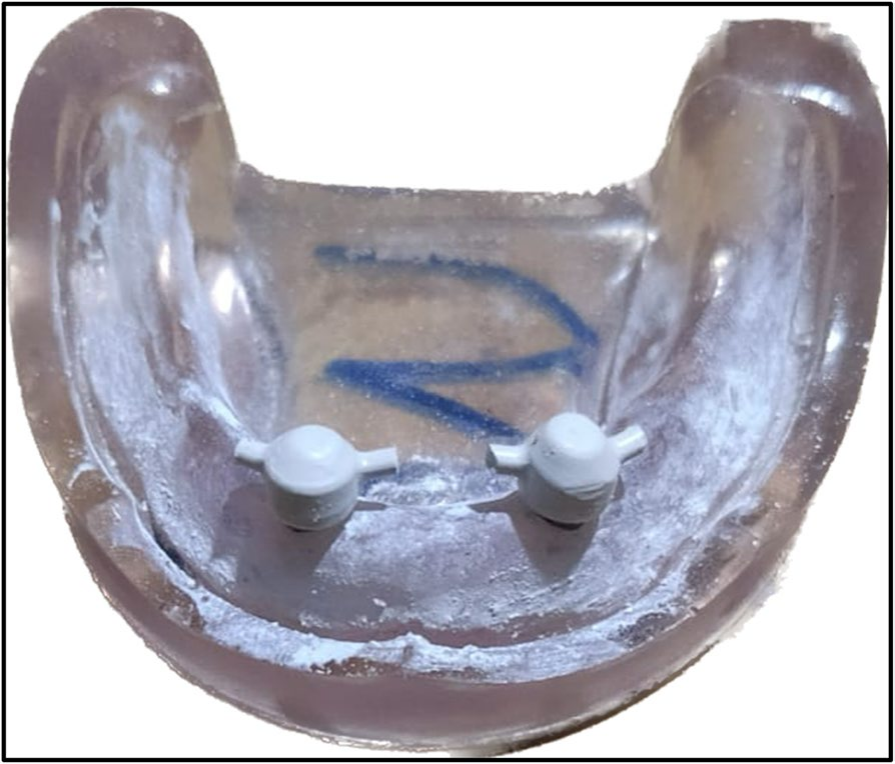
The secondary telescopic copings after milling

For the secondary coping fabrication, primary copings were first scanned, and the design of the secondary copings was established. The zirconia coping (ZrO_2_) (Katana Zirconia, HTML Plus, Japan) was designed and machined using the same program and milling equipment to simulate the crown wall conversion of the primary coping. The cobalt-chromium copings (CoCr) (CoCrW-Dental alloy, Scheftner, Germany) were milled using the same parameters as the other research groups and then milled using the CAD/CAM laser technology (Figs. 9 and 10) [23].

### Pick-up technique for final overdenture fabrication

**Fig. 11:**
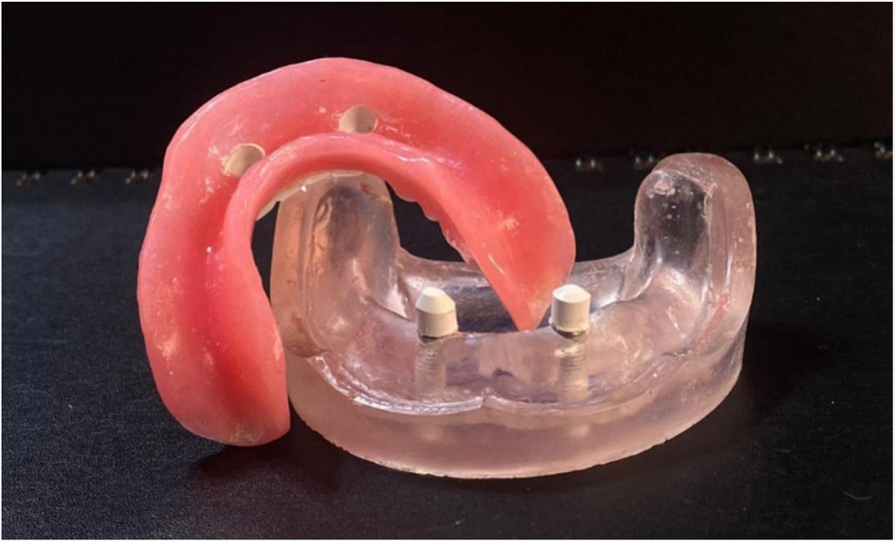
Overdenture with PEEK secondary coping

**Fig. 12:**
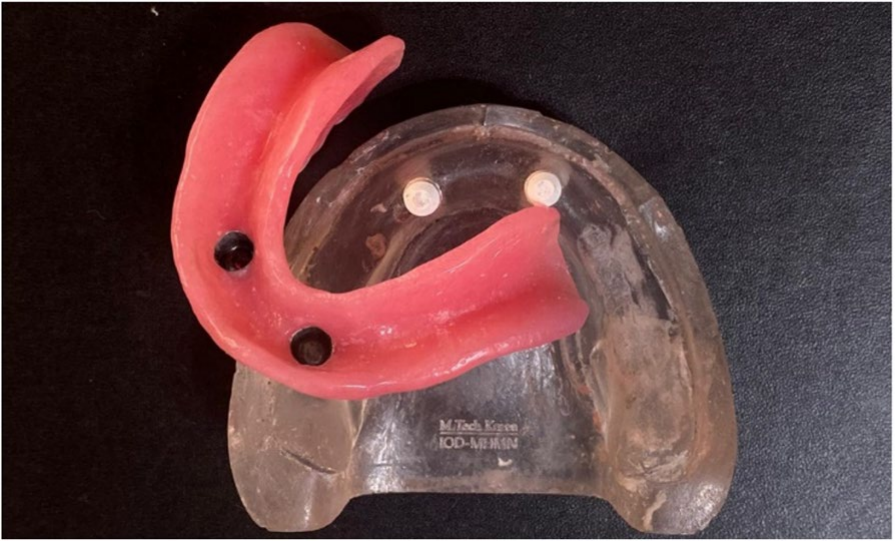
Overdenture with CoCr secondary coping

**Fig. 13:**
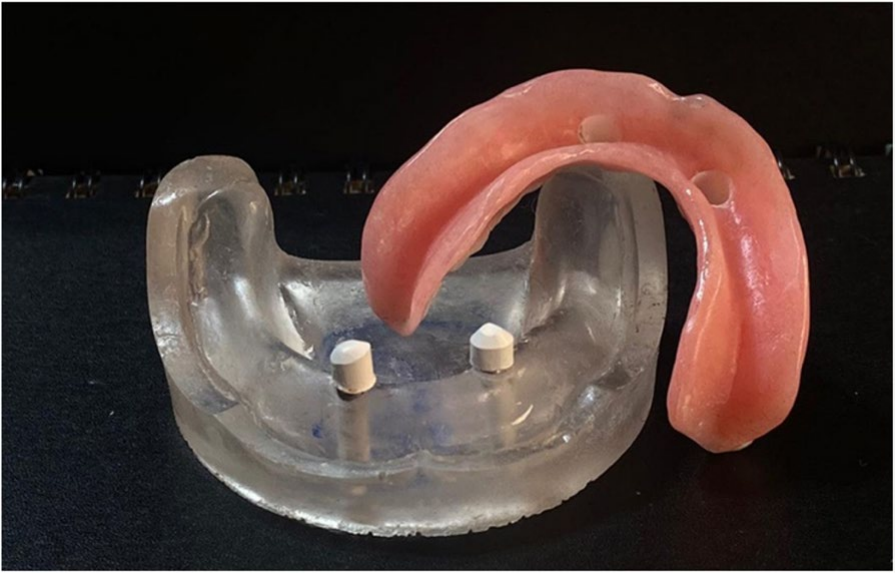
Overdenture with ZrO_2_ secondary coping

On the transparent acrylic resin model, the secondary telescopic crowns were placed over the primary crowns. Venting holes were drilled through the lingual flanges in the fitting surface of the denture. The secondary crowns were placed over the primary crown in the proper path of insertion before being picked up to the intaglio surface of the overdenture using self-cured PMMA (Acrostone acrylic material, England) (Figs. 11, 12 and 13).

### Denture preparation for testing

**Fig. 14:**
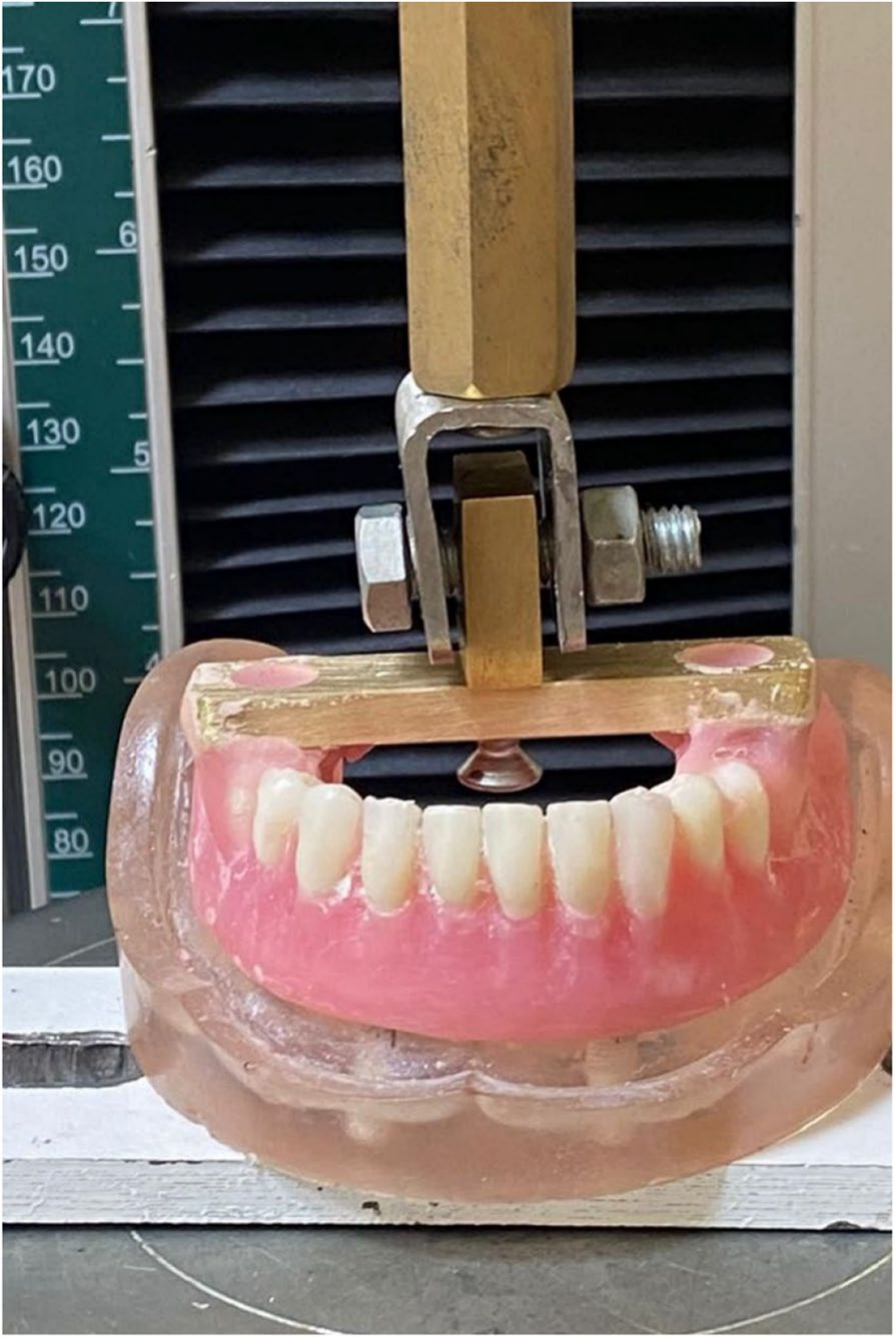
Metallic cobalt-chrome bar attachment with grasping hook in the middle of the bar was fabricated to engage the denture and facilitate the application of the tensile load

In order to facilitate the pull-off test, a metallic cobalt-chrome bar attachment with grasping hook in the middle of the bar was fabricated to engage the denture and facilitate the application of the repeated load (Fig. 14). The bar attachment was hovered in place using self-cure acrylic resin in the premolar-molar region. The models were set in a cyclic loading machine to apply repeated loads [24].

### Cyclic loading machine

**Fig. 15:**
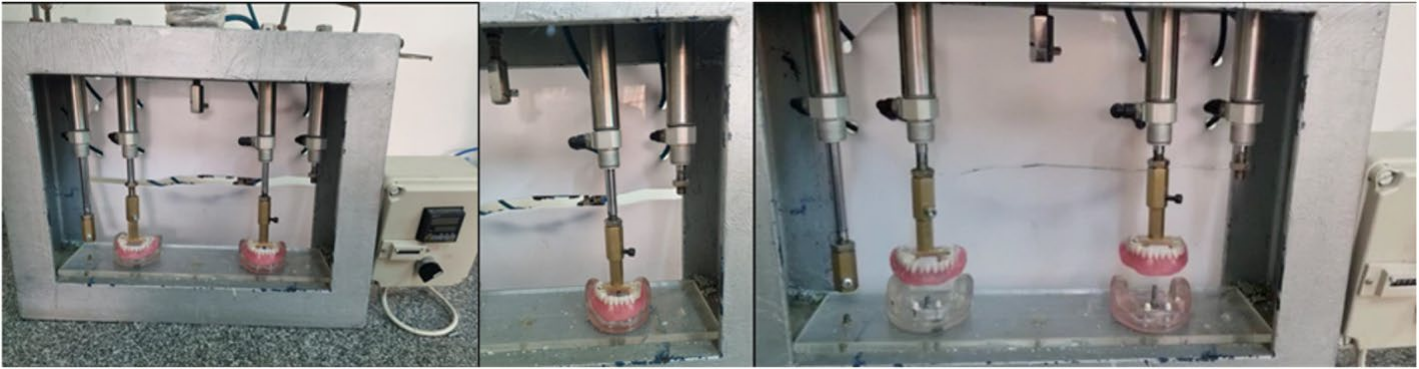
Cyclic loading machine

The cyclic loading machine was set at a speed of 30 mp and used to apply repeated insertion removal cycles at 1.000, 5.000, and 10.000 cycles, simulating nearly 10 years of function intra-orally, in which the overdenture was removed three times per day. (Fig. 15) [24, 25],

### Wear resistance testing using stereomicroscope [26]

**Fig. 16:**
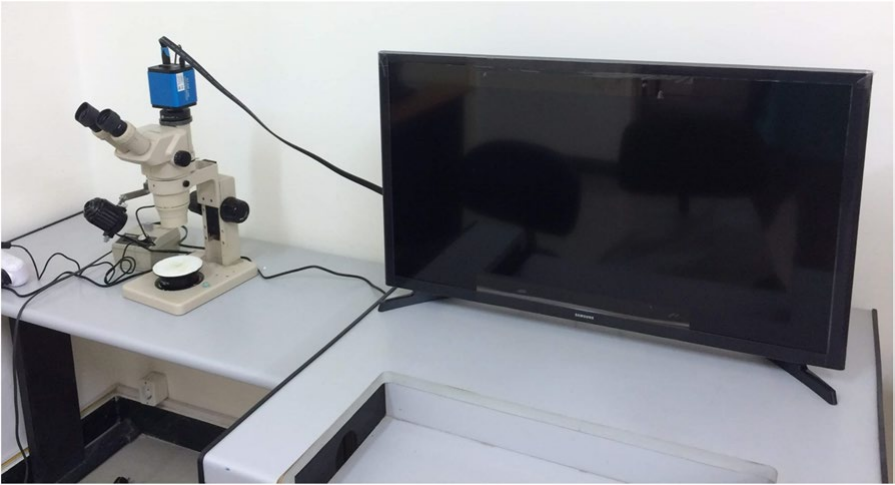
Stereomicroscope

A digital microscope (SZ1145TR, Olympus, Japan) with a built-in camera (XCAM1080PHB, ToupCam, Japan) was used to photograph the examined attachment samples. This microscope was connected to an IBM compatible personal computer (Toup View, Version 3.7). Three grooves were marked on the models (one groove placed anteriorly and two grooves on each side) to ensure accurate and repetitive placement of the models during each examination (Fig. 16).

During examination, the microscopic power of magnification was adjusted to 110x. The images of the examined attachment samples were captured at a resolution of 300 ppi, via Microsoft Office Picture Manager software and cropped to 350 × 400 pixels in order to standardize all the images of the different examined samples. A 3D image of the surface profile of each examined attachment sample was acquired via a digital image analysis system.

Every shot was taken considering the wear surface of the attachment sample parallel to the base of the used microscope used. The surface area was measured as the distance between the mesio-distal boundaries of the telescopic attachments coping.

**Fig. 17:**
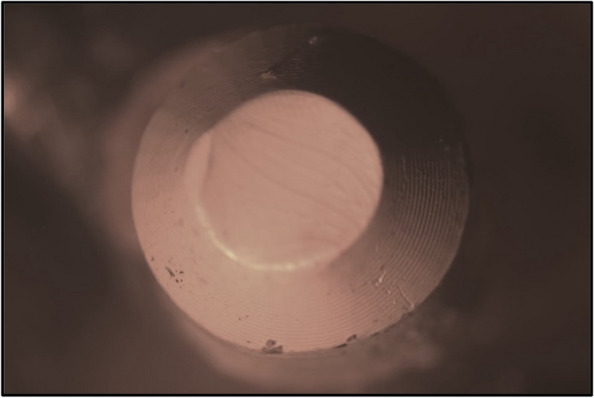
PEEK Primary coping before cyclic loading application for PEEK-PEEK group

**Fig. 18:**
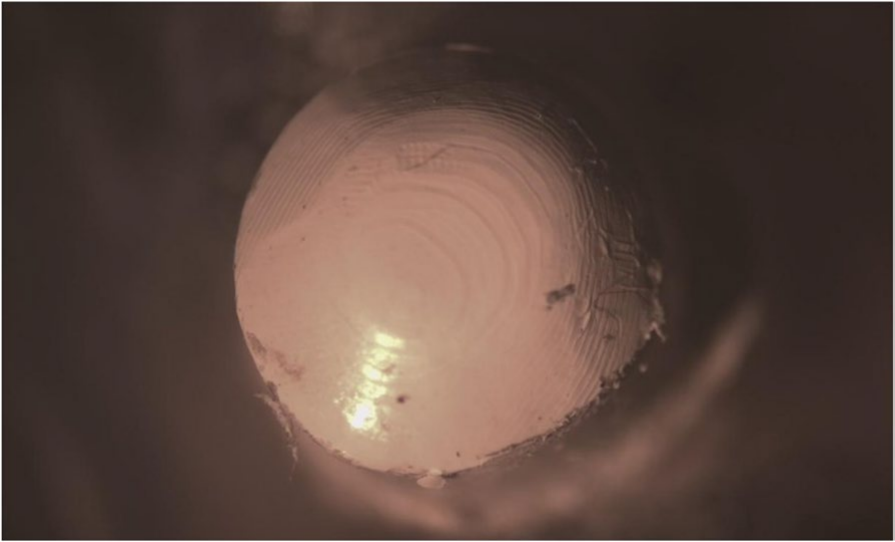
PEEK Primary coping after 10.000 cyclic loading application for PEEK-PEEK group

**Fig. 19:**
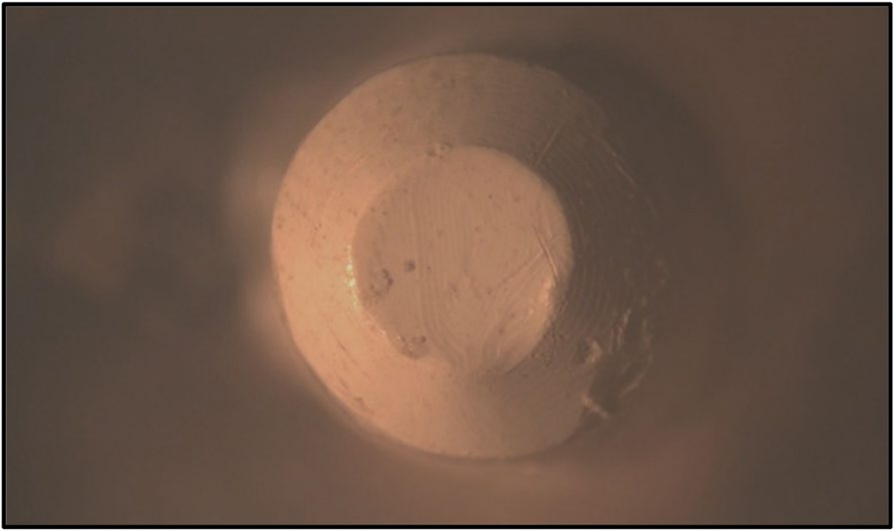
PEEK Primary coping before cyclic loading application for PEEK-ZrO_2_ group

**Fig. 20:**
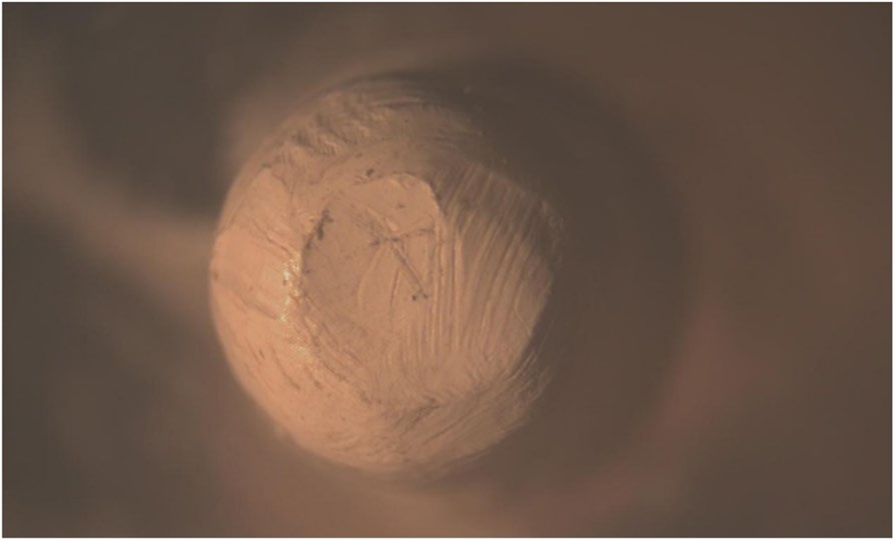
PEEK Primary coping after 10.000 cyclic loading application for PEEK-ZrO_2_ group

**Fig. 21:**
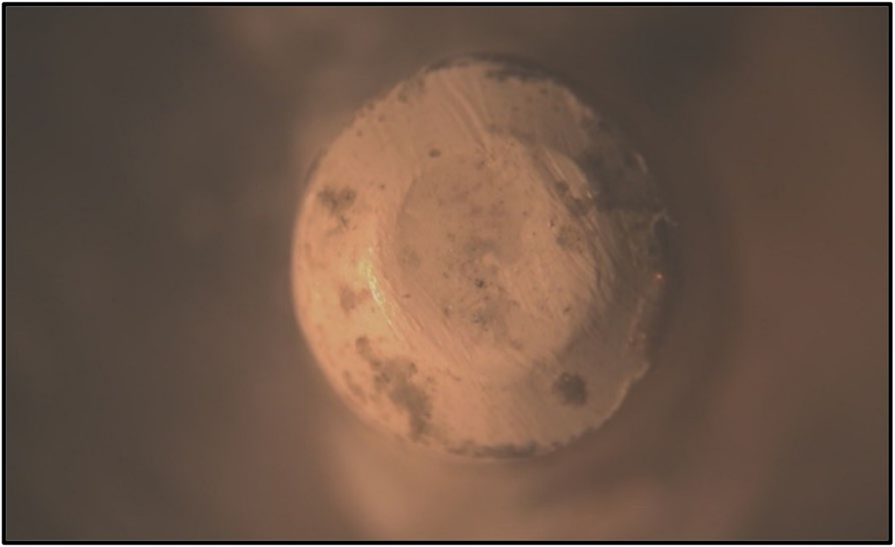
PEEK Primary coping before cyclic loading application for PEEK-CoCr group

**Fig. 22:**
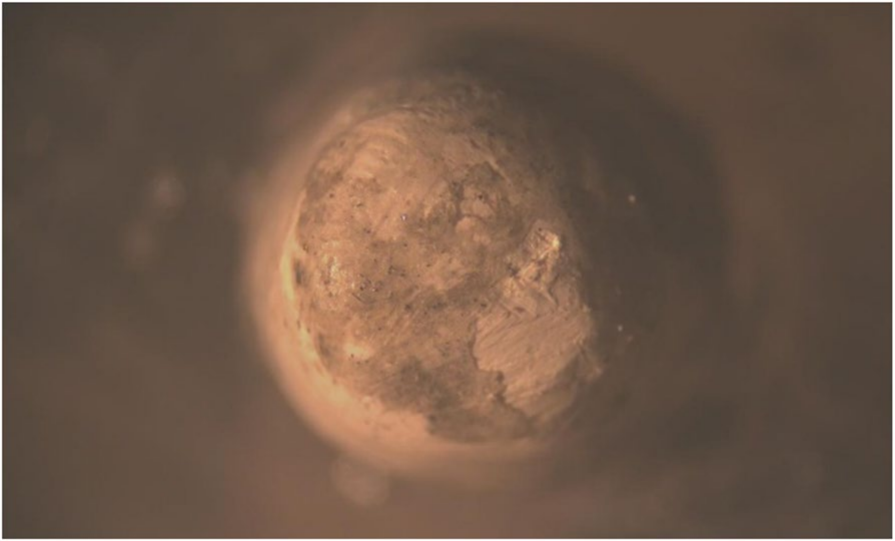
PEEK Primary coping after 10.000 cyclic loading application for PEEK-CoCr group

The primary copings of all study groups were examined under a stereomicroscope. The mesio-distal width was measured at three points, i.e., superior, middle and inferior, and the average width was calculated before the application of insertion-removal cycles and after the completion of 1.000, 5.000 and 10.000 cycles to evaluate the reduction in width and surface wear of each group (Figs. 17, 18, 19, 20, 21 and 22).

## Results

### Statistical analysis

Data were analyzed using IBM SPSS (version 29.0) and R software (version 4.3.2). The achieved results undergo evaluation at a 5% Significance Level, and a 95% Confidence interval.

- ANOVA test was used to identify significance difference among the means of more than two groups, followed by a post hoc test to determine the difference between each pair.
- Nonparametric tests were conducted via Kruskal-Walli’s tests to detect significant differences in quantitative variables between different variables.
- Repeated ANOVA test was used to detect the significance of repeated measures of the same group. Graphics were used for data visualization.

All other variables are controlled across the groups (e.g., the exact placement of the implants, and identical processing times for all the samples) to isolate the effect of the material combination on the wear resistance.

### Evaluation of surface wear

At the end of cyclic loading, dimensional changes (surface wear) of the primary copings in all the study groups were evaluated. All the materials in the study groups showed wear effects.

**Fig. 23:**
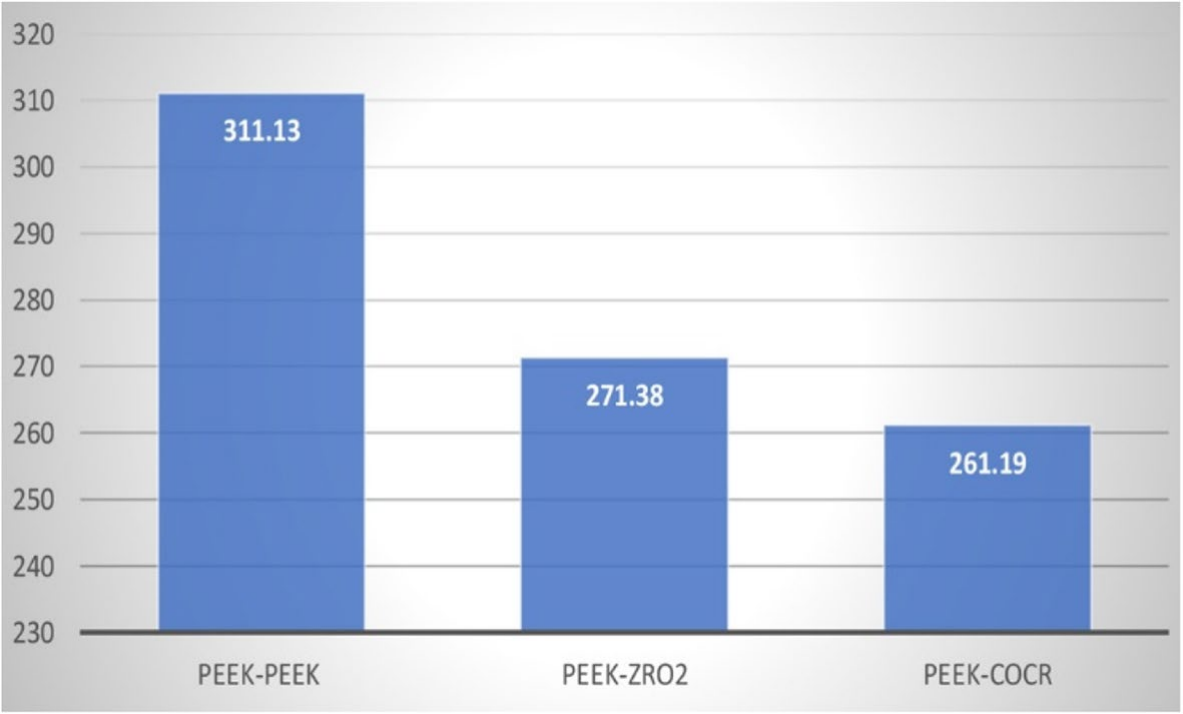
Width of primary coping of the study groups after the application of 1.000 cycles

Table (1) shows the width of the study groups after the application of repeated 1.000 insertion-removal cycles in all study groups (Fig. 23).

**Table 1:** Width of primary coping of the study groups after the application of 1.000 cycles

	**PEEK/ PEEK**		**PEEK/ZrO**_2_		**PEEK/CoCr**		**Sig.**
	**Mean**	**SD.**	**Mean**	**SD.**	**Mean**	**SD.**	
**Wear** **at 1000 cycles**	311.13	48.17	271.38	58.57	261.19	26.13	*p*<.006*
***Between groups Sig.***	*pGroup1-Group2=<.08* pGroup1-Group3=<.004* *pGroup2-Group3=<.20*						

This table shows the width of the study group after the application of repeated 1.000 insertion-removal cycles in all study groups. The mean of PEEK/PEEK group was 311.13 (± 48.17 SD), the mean of PEEK/ ZrO_2_ was 271.38 (± 58.57 SD), and the mean of PEEK/CoCr was 261.19 (± 26.13 SD)

*Significant results ≤.05. Different superscripts denote significant pairwise comparison between different groups

There was a highly statistically significant difference between the PEEK-PEEK and PEEK-CoCr, with no significant difference between PEEK-PEEK and PEEK-ZrO_2_ or between PEEK-ZrO_2_ and PEEK-CoCr.

**Fig. 24:**
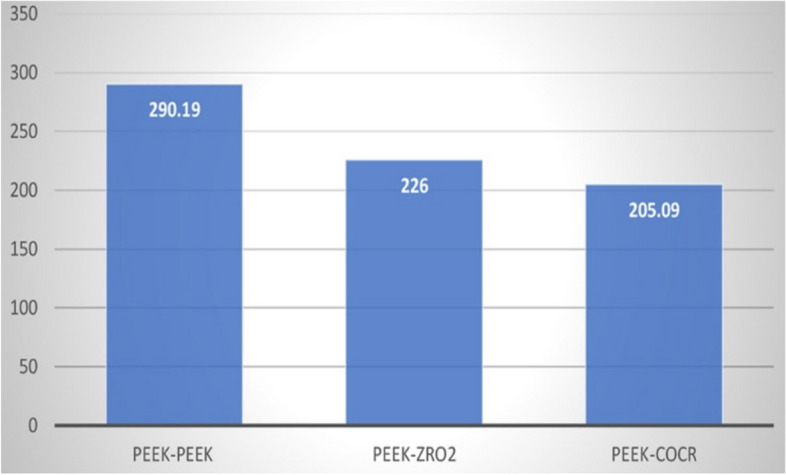
Width of primary coping of the study groups after the application of 5.000 cycles

Table (2) shows the width of the study groups after the application of 5.000 repeated insertion-removal cycles in all study groups. There was a highly statistically significant difference between the PEEK-PEEK group and the other study groups (Fig. 24).

**Table 2:** Width of primary coping of the study groups after the application of 5.000 cycles

	**PEEK/ PEEK**		**PEEK/ZrO**_2_		**PEEK/CoCr**		**Sig.**
	**Mean**	**SD.**	**Mean**	**SD.**	**Mean**	**SD.**	
**Wear** **at 5000 cycles**	290.19	32.09	226.00	47.16	205.09	16.06	*p*<.0001*
***Between groups Sig.***	pGroup1-Group2=<.013* pGroup1-Group3=<.000* *pGroup2-Group3=<.78*						

This table shows the width of the study group after the application of repeated 5.000 insertion-removal cycles in all study groups. The mean of PEEK/PEEK group was 290.19 (± 32.09 SD), the mean of PEEK/ ZrO2 was 226.00 (± 47.16 SD), and the mean of PEEK/CoCr was 205.09 (± 16.06 SD)

*Significant results ≤.05. Different superscripts denote significant pairwise comparison between different groups

**Fig. 25:**
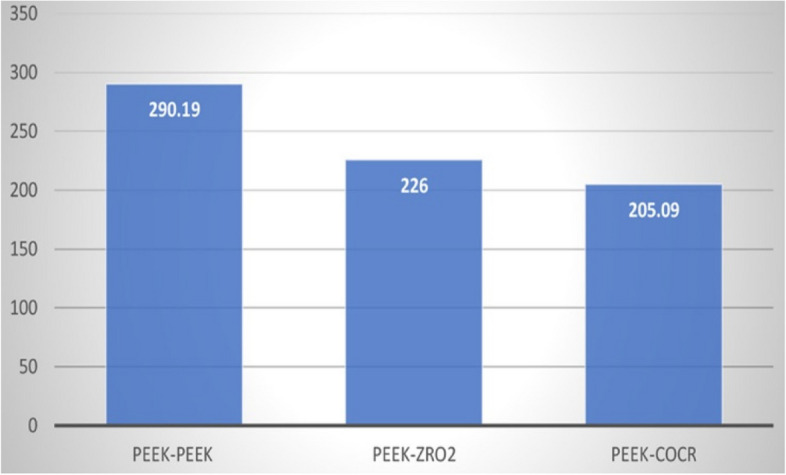
Width of primary coping of the study groups after the application of 10.000 cycles

Table (3) shows the width of the study group after the application of 10.000 repeated insertion-removal cycles in all study groups. There was a highly statistically significant difference between the PEEK-PEEK group and the other study groups (Fig. 25).

**Table 3:** Width of primary coping of the study groups after the application of 10.000 cycles

	**PEEK/ PEEK**		**PEEK/ZrO**_2_		**PEEK/CoCr**		**Sig.**
	**Mean**	**SD.**	**Mean**	**SD.**	**Mean**	**SD.**	
**Wear** **at 10.000 cycles**	277.11	27.82	177.00	26.93	163.00	21.82	*p*<.0001*
***Between groups Sig.***	pGroup1-Group2=<.001* pGroup1-Group3=<.000* *pGroup2-Group3=<1.0*						

This table shows the width of the study group after the application of repeated 10.000 insertion-removal cycles in all study groups. The mean of PEEK/PEEK group was 277.11 (± 27.82 SD), the mean of PEEK/ ZrO_2_ was 177.00 (± 26.93 SD), and the mean of PEEK/CoCr was 163.00 (± 21.82 SD)

*There was a statistical difference between the study groups

## Discussion

The present study revealed that different material combinations used for the construction of primary and secondary crowns for telescopic attachment affect the resistance of the primary coping to wear. Therefore, the null hypothesis was rejected.

The following aspects of novelty in the current work are anticipated to attract the attention of the readers. This is the first study to compare the wear resistance between three different metallic and non-metallic telescopic attachments and the changes in surface between these three different materials. Importantly, no studies in the literature have compared three material combinations in terms of wear resistance. Additionally, very few previous research has considered the use of PEEK as a primary coping [14].

The explanation for these results is the fact that the physical properties, cohesion between the materials and the ability of the materials to absorb occlusal stresses result in greater resistance to wear. In addition, the reduced surface roughness of some materials reduces friction between the contacting surfaces during the insertion and removal cycles, which might be the cause of reduced wear.

Wear-induced loss of retention represents a major clinical problem in attachment retained overdentures. Therefore, the selection of attachment type essentially depends on the material and design, which will offer the best conditions for long functional life [27, 28].

According to *Emera et al.*, following the simulation of six months of overdenture usage, significant wear was observed in all the study groups. As only the axial load was simulated, this might be the consequence of specific attachment surfaces being worn selectively over time [29].

In the present study, the wear resistance of the samples was evaluated. The PEEK-PEEK group presented the highest level of wear resistance among all the study groups. These results are consistent with those of *Shehata et al.*, who reported that PEEK telescopic crowns presented enhanced wear resistance values than CoCr telescopic crowns did. This might be due to the fact that PEEK crowns absorb occlusal stresses and wear like natural teeth do since they have a lower modulus of elasticity (4 GPa) than other standard materials, such as titanium (110 MPa) or zirconia (210 GPa) [26].

On the other hand, a study performed by *Emera et al.,* disagreed with our results, in which they reported that ZrO_2_/PEEK crowns yielded better results in the implants retained mandibular overdentures. This might be due to the low abrasion and wear potential of zirconia crowns [29].

The limitations of the following study were that only a vertical load was applied to the attachments. Other factors might affect the results such as the degree of taper of the primary coping, thermos-mechanical stimulation and salivary flow rate.

Further in vitro studies are recommended to evaluate the effect of lateral force on different types of attachments and the impact of different material combinations on the stability of the dentures to aid in the future clinical studies. Thermo-mechanical loading and fatigue testing are also recommended to validate the findings in the clinical studies.

## Conclusion

The materials used for telescopic crowns influence the absolute resistance to wear, even if the same crown design is chosen. The PEEK-PEEK combination resulted in better outcomes in terms of wear resistance, followed by PEEK-ZrO_2_. In PEEK-CoCr copings. Deposition of metal particles on the surface of the primary coping in PEEK-CoCr group was noticed.

Due to the application of a vertical load only, the reduction in width of the primary coping and surface topography was noticed only in the coronal part of the samples, whereas the remaining surface on the copings presented a reduced alteration in the surface.

## Data Availability

All datasets and materials used and/or analyzed during the current study are included in this published article.

## Abbreviations

ZrO_2_: Zirconia
PEEK: Poly-Ether-Ether-Ketone
CoCr: Cobalt-Chrome
CAD/CAM: Computer-aided-Design/Computer-aided-Manufacturing
VLC: Visible-Light-Cure-Acrylic-Resin
Dicom: Digital Imaging and Communications in Medicine
STL: Standard Triangle Language or Standard Tessellation Language
PMMA: Poly-Methyl-Methacrylate
